# Emergence of a novel FRI-type carbapenemase; *bla*FRI-12 in *Enterobacter asburiae* located on an IncR plasmid

**DOI:** 10.1007/s10096-024-04907-7

**Published:** 2024-07-24

**Authors:** Laura F. Mataseje, Florence Doualla-Bell, Ken Fakharuddin, Simon Wong, Ariane Yechouron

**Affiliations:** 1 National Microbiology Laboratory, Winnipeg, MB R3E 3R2 Canada; 2 Laboratoire de santé publique du Québec, Ste-Anne-de-Bellevue, Québec Canada; 3 Département clinique de médecine de laboratoire, Centre Hospitalier de l’Université de Montréal, Montréal, Québec Canada; 4 Service de microbiologie-infectiologie, Département de médecine spécialisée, CIUSSS du Centre-Sud de Montréal, Montréal, Québec Canada

## Abstract

Carbapenem-resistance in *Enterobacter* spp due to acquisition of mobile carbapenemases is of concern. An *Enterobacter* spp grew on ChromID CARBA medium and was positive for the mCIM carbapenemase detection assay. Susceptibility testing showed resistance to aztreonam and reduced susceptibility to imipenem. Conventional PCR using FRI primers detected a *bla*_FRI_ gene. Whole genome sequencing reveled a new variant; *bla*_FRI−12_ was closest in sequence to *bla*_FRI−5_ differing by 13 amino acids and was found on a unique 110Kb IncR plasmid. Given the intrinsic nature of *Enterobacter* spp. to be carbapenem non-susceptible, *bla*FRI-types may be under reported globally.

**Supplementary Information:**

The online version contains supplementary material available at 10.1007/s10096-024-04907-7.

## Introduction

A report on the global burden of bacterial antimicrobial resistance (AMR) from 2019 was published and indicated *Enterobacter* species were in the top 9 bacterial species associated with global deaths attributable to and associated with bacterial AMR in infections [1]. In 2020, *Enterobacter* species were found to be the top species to produce carbapenemases in Canadian hospitals, just behind *Escherichia coli* [2]. *Enterobacter cloacae complex* (ECC) are common nosocomial pathogens and often associated with multi-drug resistance including the carbapenems [3]. Though carbapenem resistance in ECC may be related to overexpression of a β-lactamase or AmpCs combined with decreased outer membrane permeability, it is the production of carbapenemases harboured on mobile genetic elements that are most concerning [3]. Minor class A carbapenemases, such as FRI-type are emerging. The first French imipenemase (FRI) variant was reported in 2015 isolated from *Enterobacter cloacae* [4]. Since then limited reports on FRI-type carbapenemases have been described and include those from the UK (FRI-2) [5], Germany (FRI-3) [6], Japan (FRI-4, FRI-5, FRI-7-11) [7–9] China (FRI-11) [10] and Canada (FRI-6, FRI-8) [11, 12]. Here we describe a new FRI-type isolated from an *Enterobacter asburiae* in Canada.

## Materials and methods

### Strain identification phenotypic and molecular testing

*Enterobacter spp* N22-01531 was suspected to harbour a carbapenemase due to growth on ChromID CARBA medium from a rectal swab. Initial susceptibility testing determined that the isolate was resistant to ertapenem (2 mg/L) and imipenem (4 mg/L) and intermediate to meropenem (2 mg/L) by broth microdilution using CLSI guidelines. Phenotypic testing was conducted using modified carbapenem inactivation method (mCIM) using CLSI guidelines. Following a positive mCIM test this isolate was sent to the National Microbiology Laboratory (NML) for further testing. Additional phenotypic testing for carbapenemase production was done using the Rosco Neo-Rapid Carb Kit and β CARBA kit. Conventional PCR containing FRI specific primers (FRI-F-U1: 5’-TAAACTCAGCTATTCCAGGC-3’ and FRI-R-U2: 5’-ACAGGTGCCTGTTTTATCGCC-3’) was conducted yielding a positive result [11]. Antimicrobials susceptibility testing was done using the commercial Sensititre™ panel from Thermo Scientific™.

### Whole genome sequencing

Once confirmed positive by FRI primers the isolate was sent for whole genome sequencing (WGS). DNA was extracted using Qiagen DNeasy kits (Qiagen, Toronto, Canada) and sequenced on an Illumina NextSeq™ platform using Nextera XT libraries. MinION sequencing (Nanopore Technologies, Oxford, UK) was conducted using the rapid kit (SQK-RBK 004) on R9.4.1 flowcells and run on Guppy 6.3.7 using the super accurate basecalling model. De novo hybrid assemblies were done using Unicycler 0.4.7 [13]. Analysis was conducted using the StarAMR pipeline (https://github.com/phac-nml/staramr) for resistance gene detection, plasmid detection and multilocus sequence typing. Analysis of the genome using FastANI (https://github.com/ParBLiSS/FastANI) was used to determine species. Data for this project was deposited on NCBI under BioProject PRJNA865257.

## Results and discussion

A 91-year-old patient presenting with severe neuro-cognitive disorders entered the emergency department after suffering injuries from a fall during 2021. She was hospitalized for three weeks where she received a 2 day course of tazobactam. She had no known history of travel or acute infections or stay in palliative care. She was screened for CPE following a suspected outbreak of KPC on the floor where she was hospitalized. The result of her mCIM came back positive and no carbapenemase genes were found following testing for NDM, KPC, OXA-48-like, IMP, VIM, GES, NMC and IMI. The patient was moved to a residential and long-term care center and in June 2022 and tested positive for mCIM following routine screening practices. At this time PCR targets for FRI had been developed due to a separate FRI case reported in Canada [11]. The CPE isolate (N22-01531) was confirmed to be FRI-positive as were all retrospective isolates from this patient (data not shown).

Susceptibility testing showed resistance to aztreonam only. The isolate was susceptible to meropenem and ertapenem while intermediate to imipenem (Table 1). Similarly to another report [14] novel β-lactam/ β-lactamase inhibitor combinations were effective against our clinical FRI containing isolate. As previously observed for FRI-6 [11], phenotypic detection of carbapenemase using mCIM and Rosco Neo-Rapid tests exhibited positive results while β CARBA was negative. Concerning FRI-8 [12], only mCIM detected FRI production. Similar discrepancies between phenotypic assays were reported concerning other FRI-variants; indeed, while β CARBA didn’t detect FRI-1 [15], mCIM was positive for multiple variants (FRI-8, FRI-4, FRI-2, FRI-5) [5, 7, 8, 16] and carba NP showed variable results (negative for FRI-8, inconsistent for FRI-11 [9] and positive for FRI-4, FRI-5, FRI-2, FRI-1 [4, 5, 7, 8]). Interestingly, mCIM was positive for all FRI-variants positive, however, this test may exhibit false positive results with *E. cloacae* due to AmpC over production [17, 18]. Indeed, further work needs to be done in FRI-type carbapenemase to avoid over testing *E. cloacae* non-carbapenemase producers using mCIM.

**Table 1:** Antimicrobial susceptibilities (mg/L) for strains tested in this report

Sensititre CAN1MSF	E. asburiae N22-01531		E.coli TOP10 (pFRI-12TF)a		E.coli TOP10 (pblaFRI-12)^b^		E.coli TOP 10	
amikacin	<=8	S	<=8	S	<=8	S	<=8	S
aztreonam	**16**	R	**> 16**	R	**> 16**	R	<=1	S
cefepime	<=1	S	<=1	S	<=1	S	<=1	S
ceftazidime	<=4	S	<=4	S	<=4	S	<=4	S
ceftriaxone	<=1	S	**4**	R	<=1	S	<=1	S
ceftazidime/avibactam	<=4	S	<=4	S	<=4	S	<=4	S
ciprofloxacin	<=0.06	S	<=0.06	S	<=0.06	S	<=0.06	S
colistin	<=1	I	<=1	I	<=1	I	<=1	I
doxycycline	<=4	S	<=4	S	<=4	S	<=4	S
ertapenem	<=0.25	S	**> 2**	R	<=0.25	S	<=0.25	S
ceftolozone/tazobactam	<=1	S	**8**	R	<=1	S	<=1	S
gentamicin	<=2	S	<=2	S	<=2	S	<=2	S
imipenem/relebactam	2	S	<=1	S	<=1	S	<=1	S
levofloxacin	<=0.5	S	<=0.5	S	<=0.5	S	<=0.5	S
meropenem	0.25	S	**4**	R	<=0.06	S	<=0.06	S
meropenem/vaborbactam	<=1	S	<=1	S	<=1	S	<=1	S
minocycline	<=4	S	<=4	S	<=4	S	<=4	S
piperacillin/tazobactam	<=8	S	**> 64**	R	**> 64**	R	<=8	S
plazomicin	<=1	S	<=1	S	<=1	S	<=1	S
tigecycline	<=0.5	S	<=0.5	S	<=0.5	S	<=0.5	S
tobramycin	<=2	S	<=2	S	<=2	S	<=2	S
trimethoprim/ sulfamethoxazole	<=2	S	<=2	S	<=2	S	<=2	S
**Etest**								
cefoxitin	6	S	8	S	8	S	< 0.25	S
Imipenem	2	I	4	R	0.5	S	0.125	S

Bold formating indicates resistant phenotype according to CLSI (M100:2023). S-susceptible; I-intermediate; R-resistant

^a^E.coli TOP10 electrotransformed with pFRI-12

^b^*bla*_FRI−12_ and upstream region amplified from N22-01531 with primers FRI-12 A (forward-GATTTCTCATTGTATACCAACC) and FRI-12B (reverse-TGGCGGACATTTCATGGCGC), cloned into pCR21.TOPO (ThermoFisher Scientific). pCR2.TOPO contains an ampicillin resistance gene (*bla*_TEM−1_) and kanamycin resistance gene (aphII)

Data from WGS revealed the presence of an *Enterobacter asburiae* harbouring the novel FRI-variant *bla*_FRI−12 (_reference sequence number NG_081789.1). FRI-12 had the closest amino acid identity to FRI-5 differing by 13 amino acids (95.6% identity) (Supplementary Fig. 1).

**Fig. 1:**
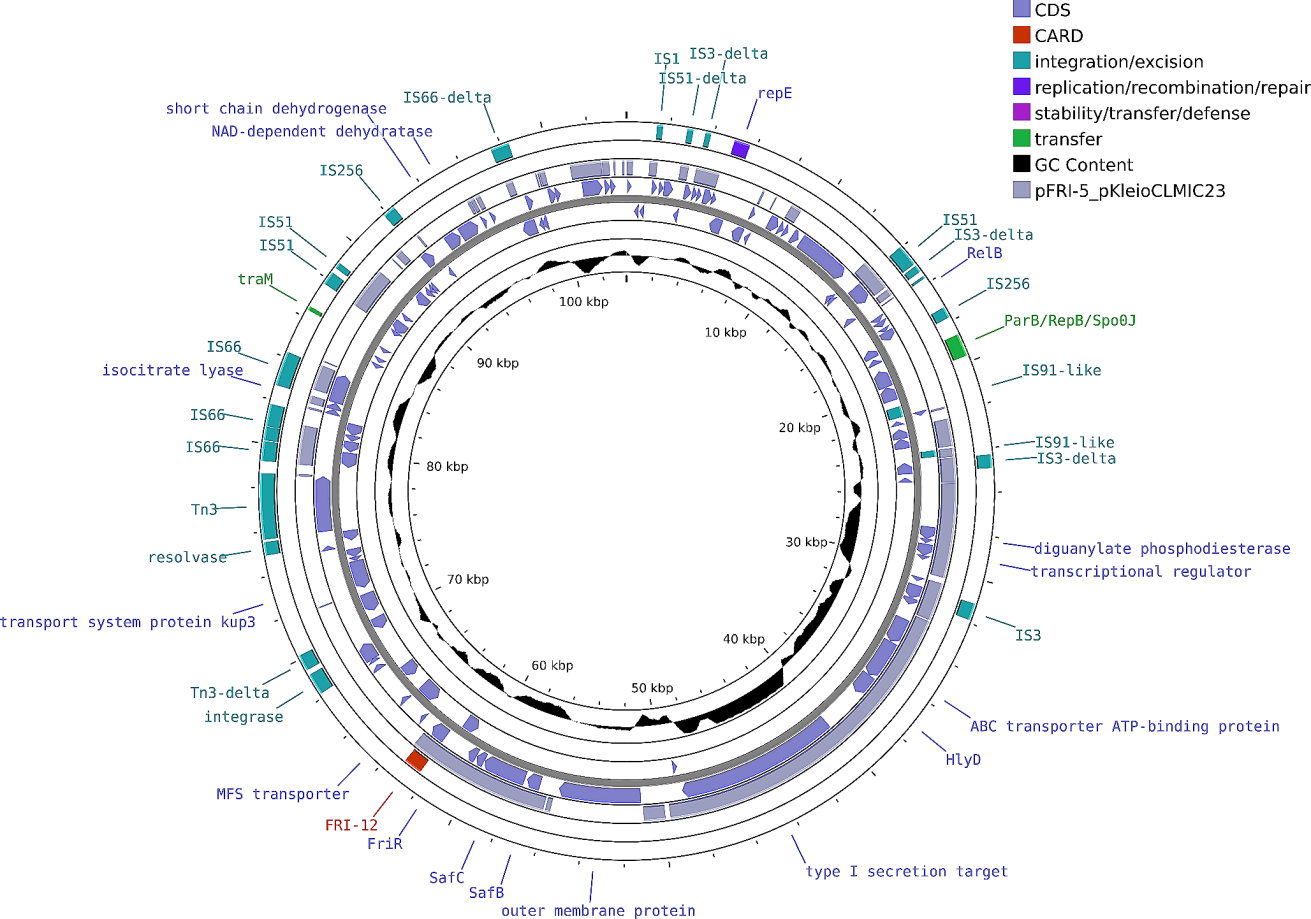
FRI-12 plasmid generated with Proksee. Innermost two tracks represent forward and reverse open reading frames of pN22-01531. Third track represents BLASTn of FRI-12 plasmid to its closest related plasmid (pFRI-5). Outer two tracks are annotations generated by CARD RGI and mobileOG databases

The *bla*_FRI−12_ gene was found on a 104 Kb IncR plasmid (pN22-01531, Fig. 1). The closest hit to a known plasmid was pKeioCLMIC23 harbouring *bla*_FRI−5_ (accession number AP028423.1) which shared 44% coverage at 95% identity. Indeed, when all other FRI plasmids were BLAST against pN22-01531only 5–17% coverage was observed. Previous reports have shown all *bla*_FRI−type_ genes are harboured on plasmids containing the replicon IncFll-type [10]. However, some of these (FLC-1, FRI-2, FRI-4, FRI-9 and FRI-11) are multi-replicon plasmids that also contain IncR [10]. Interestingly, the IncFII-type FRI-5 plasmid (pKeioCLMIC23), which shared the closest identity to our FRI-12 plasmid did not contain the IncR replicon as observed in other FRI-variants. The alignment of FRI surrounding regions in different variants highlights a diverse arrangement of coding sequences (Supplementary Fig. 2). The FRI-12 surrounding region was most similar to FRI-5 where we observed a common 7 Kb of shared sequence representing FRI-12, FriR (a LysR transcriptional regulator), SafC and SafB (components of a *saf* operon for pili functionality). No additional resistance genes were found on the FRI-12 IncR plasmid.

Similar to our previous reports [11, 12], we were able to successfully transform pN22-01531 into *Escherichia coli* TOP10 (labeled pFRI-12TF), however, conjugation experiments with *Escherichia coli* J53AzR were not successful. Other than with FRI-6 [11] this phenomenon is not uncommon in FRI-type plasmids. We cloned *bla*_FRI−12_ into pCR2.1TOPO and subsequently transformed this plasmid into *E. coli* TOP10 (pblaFRI-12) to test its antimicrobial susceptibility (Table 1). The transformant showed higher MICs than the clinical strain to aztreonam, ceftriaxone, cefoxitin, ertapenem, meropenem, imipenem, ceftolozone/tazobactam and piperacillin/tazobactam. However, the cloned *bla*_FRI−12_ gene showed higher MIC to piperacillin/tazobactam only. Further work will need to be conducted on FRI-12 plasmid copy number and gene expression to establish if they affect MIC to selected antimicrobials.

To our knowledge, this is the first published report of *bla*_FRI−12_ identified globally. This study highlights the benefits of phenotypic screening of carbapenemases in *Enterobacter* sp. isolates exhibiting non-susceptible MICs to carbapenems despite susceptibility to cephalosporins.

## Electronic supplementary material

Below is the link to the electronic supplementary material.

Supplementary Figure 1: Amino Acid alignment of FRI-variants and phylogenetic tree showing relatedness among FRI-type sequences. Conserved Ambler class A regions; active site motifs ^70^SXXK^73^, ^130^SDN^132^, ^166^EXXXN^170^ and ^234^KTG^236^ and cysteine residues C^69^ and C^238^ are shown [6]

Supplementary Figure 2: Schematic representation of FRI region among all FRI variants. Black arrows are hypothetical proteins. All other arrows are labelled

## Data Availability

The datasets generated during and/or analysed during the current study are available in the NCBI repository under BioProject PRJNA865257.
