# Supplementary figures and images for: Emergence of a novel FRI-type carbapenemase; blaFRI-12 in Enterobacter asburiae located on an IncR plasmid
Source: Eur J Clin Microbiol Infect Dis. 2024 Jul 24;43(10):2047–51. doi: 10.1007/s10096-024-04907-7 (PMC11405460; doi:10.1007/s10096-024-04907-7)

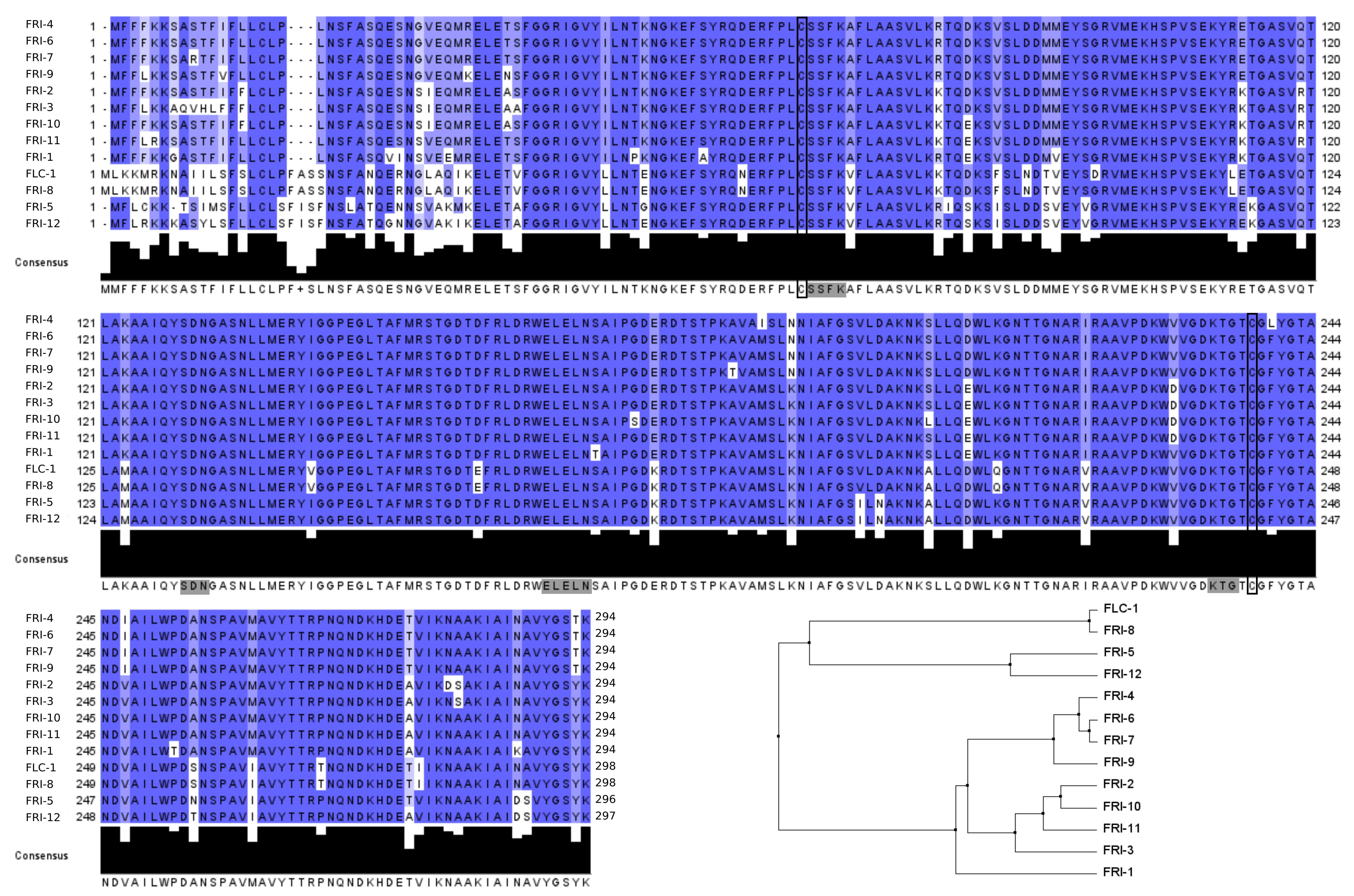

Supplement: Supplementary file 1 — Supplementary Figure 1: Amino Acid alignment of FRI-variants and phylogenetic tree showing relatedness among FRI-type sequences. Conserved Ambler class A regions; active site motifs 70SXXK73, 130SDN132, 166EXXXN170 and 234KTG236 and cysteine residues C69 and C238 are shown [6] [file 10096_2024_4907_MOESM1_ESM.png]
